# Bortezomib Treatment Produces Nocifensive Behavior and Changes in the Expression of TRPV1, CGRP, and Substance P in the Rat DRG, Spinal Cord, and Sciatic Nerve

**DOI:** 10.1155/2014/180428

**Published:** 2014-04-27

**Authors:** M. Quartu, V. A. Carozzi, S. G. Dorsey, M. P. Serra, L. Poddighe, C. Picci, M. Boi, T. Melis, M. Del Fiacco, C. Meregalli, A. Chiorazzi, C. L. Renn, G. Cavaletti, P. Marmiroli

**Affiliations:** ^1^Department of Biomedical Sciences, Section of Cytomorphology, University of Cagliari, 09042 Monserrato, Italy; ^2^Department of Surgery and Translational Medicine, University of Milano-Bicocca, 20900 Monza, Italy; ^3^Department of Organizational Systems and Adult Health, University of Maryland School of Nursing, Baltimore, MD 21201, USA; ^4^Program in Neuroscience, University of Maryland, Baltimore, MD, USA

## Abstract

To investigate neurochemical changes associated with bortezomib-induced painful peripheral neuropathy (PN), we examined the effects of a single-dose intravenous administration of bortezomib and a well-established “chronic” schedule in a rat model of bortezomib-induced PN. The TRPV1 channel and sensory neuropeptides CGRP and substance P (SP) were studied in L4-L5 dorsal root ganglia (DRGs), spinal cord, and sciatic nerve. Behavioral measures, performed at the end of the chronic bortezomib treatment, confirmed a reduction of mechanical nociceptive threshold, whereas no difference occurred in thermal withdrawal latency. Western blot analysis showed a relative increase of TRPV1 in DRG and spinal cord after both acute and chronic bortezomib administration. Reverse transcriptase-polymerase chain reaction revealed a decrease of TRPV1 and CGRP mRNA relative levels after chronic treatment. Immunohistochemistry showed that in the DRGs, TRPV1-, CGRP-, and SP-immunoreactive neurons were mostly small- and medium-sized and the proportion of TRPV1- and CGRP-labeled neurons increased after treatment. A bortezomib-induced increase in density of TRPV1- and CGRP-immunoreactive innervation in the dorsal horn was also observed. Our findings show that bortezomib-treatment selectively affects subsets of DRG neurons likely involved in the processing of nociceptive stimuli and that neurochemical changes may contribute to development and persistence of pain in bortezomib-induced PN.

## 1. Introduction

Bortezomib (BTZ), a dipeptidyl boronic acid, is an anticancer drug mostly used in the treatment of multiple myeloma [1–4] and also in solid tumor treatment [5–7]. BTZ is a selective, reversible inhibitor of the ubiquitin-dependent proteasome system, which is the main intracellular pathway complex controlling regulated protein degradation [8–10]. BTZ selectively blocks the chymotrypsin-like proteasomal activity residing within the 26S core complex, thus impairing protein turnover and triggering a cascade of events affecting cell proliferation, cell adhesion, and angiogenesis [11–13]. This leads to cancer cell cycle arrest and apoptosis [14–16]. A distinctive feature of BTZ action is the stabilization of the intracellular inhibitor kappa B (I*κ*B) and the inhibition of nuclear factor-kappa B (NF-*κ*B) activation, with subsequent downregulation of proteins that promote cell division and proliferation [12]. These effects are most evident in malignant cells, although normal cells are also affected [13].

One of the main dose-limiting side effects in BTZ therapy (particularly when the drug is delivered intravenously) is the development of a painful, sensory peripheral neuropathy, associated with symptoms such as numbness and tingling, that occurs in a distal stocking-and-glove pattern [17, 18] and is characterized by a marked dysfunction of all fiber types in sensory nerves [17, 19], also extending into areas of the skin that are not perceived as painful [19]. Severe neuropathic pain frequently develops after the first treatment cycle [17, 20] leading to a long-term decreased quality of life [21] and often to BTZ dose modification or discontinuation [22]. Careful follow-up psychophysical studies showed that pain intensity and impairment in sensory function could persist for more than one year after therapy withdrawal [1, 19].

Experimental studies indicate that both distal peripheral nerves and dorsal root ganglia (DRGs) are targets of BTZ-induced neurotoxicity [23–26]. We have recently developed and characterized an* in vivo *rat model of BTZ-induced painful sensory neurotoxicity designed to mimic the typical long-term clinical use of the drug [27–29]. In this model, the hallmark of BTZ-induced neurotoxicity was the development of peripheral nerve axonopathy, occurring predominantly in small myelinated (Adelta fibers) and unmyelinated axons (C fibers), with occasional involvement of large myelinated axons (Aalpha/beta fibers). By contrast, no evident morphological changes occurred in DRG neurons, whereas satellite cells were mildly affected [28, 29]. The sensory behavioral assessment showed the onset of mechanical allodynia, whereas thermal perception did not change [29].

The transient receptor potential vanilloid type-1 (TRPV1) is a nonselective cation channel expressed in primary sensory neurons, where it plays an important role in transmission and modulation of nociceptive sensations [30, 31]. TRPV1 is present on polymodal nociceptors and is considered a molecular integrator that can be activated and/or sensitized by noxious heat, protons, and other endogenous compounds released following tissue injury [30]. Besides its widely recognized role as mediator of thermal nociception [32], several lines of evidence indicate that, at both peripheral and central level, TRPV1 may also contribute to mechanotransmission, particularly after the occurrence of an injury see [33–37] and the references therein). DRG neurons comprise large-sized neuron group (about 40%), which give rise to myelinated axons, and small- and medium-sized neurons that can be further subdivided into neuropeptidergic and nonpeptidergic groups. Calcitonin gene-related peptide (CGRP) is the best marker for the neuropeptidergic subpopulation (about 40% of cells), comprising mostly small neurons with unmyelinated axons (C fibers) and innervating mainly polymodal nociceptors [38]. In this neuronal category also fall most of the substance P- (SP-) expressing DRG cells. CGRP and SP are also expressed by a group of medium-sized cells with finely myelinated (Adelta) axons, most of which are nociceptors of the high-threshold mechanoreceptor type [38]. TRPV1 activation causes neuronal depolarization, which leads to pain and release of sensory neuropeptides such as CGRP and SP from peripheral and central nerve terminals. The neuropeptides activate their effector cell receptors and enhance the sensitization of nociceptors [39].

The purpose of this study was to determine if the BTZ-induced axonopathy, affecting mainly Adelta and C fibers, though not accompanied by evident changes in DRG neuronal morphology, is matched by changes in neurochemistry of polymodal nociceptors. Thus, we examined whether intravenous BTZ administration, either as a single dose or on a “chronic” schedule, affects the expression of TRPV1, CGRP, and SP in lumbar DRGs, spinal cord, and sciatic nerve. We found that BTZ-treatment affects the expression of TRPV1 and CGRP in both DRG and spinal cord inducing changes in their expression and affecting the relative percent frequency of TRPV1- and CGRP-immunolabeled DRG neurons that are likely to be polymodal nociceptors. Taken together, these findings will increase our understanding of the mechanisms of nociceptive symptoms associated with BTZ-induced sensory neuropathy and may lead to the development of new targeted therapies.

## 2. Materials and Methods

### 2.1. Animals

A total of 80 adult female Wistar rats (Harlan Italy, Correzzana, Italy), weighing 175–200 g at the beginning of the experiment, were used for the study. Animals were housed in a limited access animal facility with controlled room temperature (22 ± 2°C) and relative humidity (55 ± 10%) and under an artificial 12 h light/dark cycle (light 7 a.m.–7 p.m.). The care and husbandry of the animals were in conformity with the institutional guidelines in compliance with national (D.L. n.116, Gazzetta Ufficiale della Repubblica Italiana, Additional 40, February 18, 1992, and subsequent modifications) and international laws and policies (EEC Council Directive 86/609, OJ L 358, 1, December 12, 1987; Guide for the Care and Use of Laboratory Animals, US National Research Council, 8th ed., 2011). The experimental plan was examined and approved by the Ethics Committee of the University of Milano-Bicocca.

### 2.2. Drug Administration and Euthanasia

Bortezomib (LC Laboratories, Woburn, MA) was dissolved in 5% Tween 80, 5% ethanol, and 90% sterile saline. Animals were divided into two experimental groups. One group of 40 rats was treated with bortezomib by tail vein injection. Eighteen animals received BTZ 0.20 mg/kg as single dose (“acute” schedule) and 22 animals underwent an 8-week period of BTZ administration (“chronic” schedule, 0.20 mg/kg/day three times/week; see [28]). The remaining 40 animals were left untreated and used as controls for the 2 time points of examination. Euthanasia for tissue harvesting was done under deep xylazine/ketamine anesthesia 1 hour after the single BTZ dose in the “acute” experimental schedule and one day after the last BTZ administration in the “chronic” experimental schedule.

### 2.3. General Toxicity

The general condition of treated animals was assessed daily and body weight of the chronically treated rats was measured twice/week to monitor drug toxicity and for drug dose calculation. Animals with evident distress or remarkable weight loss were carefully examined by a certified veterinarian experienced with* in vivo* studies for possible withdrawal from the study for humane reasons.

### 2.4. Neurotoxicity and Behavioral Measures

Neurophysiological assessment and behavioral measures were performed before the beginning of the treatment period (baseline values) and at the end of the 8-week BTZ-treatment, as previously described by Meregalli et al. in the same animal model [28, 29]. Briefly, the caudal nerve conduction velocity (NCV) was assessed using an electromyographic instrument (Myto2 ABN Neuro, Firenze, Italy) by placing recording ring electrodes distally on the tail and stimulating ring electrodes 5 and 10 cm proximal to the recording points.

To evaluate the presence of chemotherapy-induced allodynia, the mechanical nociceptive threshold was measured using the Dynamic Plantar Aesthesiometer (Ugo Basile Biological Instruments, Comerio, Italy).

Two hours after Dynamic test evaluation, the response to noxious thermal stimulus was determined using a Plantar Test Instrument (Hargreaves' method; Ugo Basile Biological Instruments, Comerio, Italy).

### 2.5. Sampling

Immediately after sacrifice, the L4-L6 DRGs with the corresponding spinal cord segments and the sciatic nerves were rapidly dissected out and either frozen at −80°C for western blot and RT-PCR analyses or fixed by immersion in freshly prepared 4% phosphate-buffered paraformaldehyde, pH 7.3, for 4–6 h at 4°C, and then rinsed overnight in 0.1 M phosphate buffer (PB), pH 7.3, containing 20% sucrose for immunohistochemistry. After sucrose infiltration, samples were embedded in Optimal Cutting Temperature (OCT) medium for cryostat sectioning.

Specimens were also harvested and processed for light microscope neuropathological evaluation of resin-embedded semithin (1 *μ*m thick) sections, according to the previously described protocol [28, 29, 40–48].

### 2.6. Protein and RNA Extraction

A total of 32 DRGs and spinal cord segments were analyzed: 12 from the acutely treated group (6 BTZ-treated and 6 controls) and 20 from the chronically treated group (10 BTZ-treated and 10 controls). Total protein and RNA from DRGs and spinal cord tissue homogenates were extracted by the TRIzol method (Invitrogen, Carlsbad, CA) (1 mL/100 mg of tissue), followed by incubation in chloroform (200 *μ*L/mL) for 3 min at room temperature (RT). After centrifugation at 5000 rpm for 15 min at 4°C protein and RNA were separated into an upper aqueous phase and a lower organic phase, respectively.

The aqueous phase was transferred to new vials, incubated in isopropyl alcohol (0.5 mL/mL) for 10 min at RT, and then centrifuged at 12,000 g at 4°C for 10 min to allow RNA precipitation. The RNA pellet obtained was washed in 75% EtOH in RNase-free diethyl pyrocarbonate (DEPC) water (1 mL/L). Samples were then centrifuged at 7,500 g for 5 min at 4°C. Following complete elimination of EtOH, the pellet was dried by means of a speed-vac, resuspended in 100 *μ*L of DEPC water, and stored at −80°C until RT-PCR analysis.

The organic phase was incubated in absolute ethanol (EtOH) (300 *μ*L/mL) for 2-3 min at RT followed by centrifugation at 2,000 g for 5 min at 4°C. The supernatant was then combined with isopropyl alcohol (1.5 mL/mL), incubated for 10 min at RT, and then centrifuged at 12,000 g for 15 min at 4°C. The supernatant was withdrawn and the protein pellet was washed three times in a solution of 0.3 M guanidine-HCl (IBI scientific, Kapp Court, Peosta, IA, USA) in 95% EtOH (2 mL/mL) for 20 min at RT and centrifuged at 7500 g, for 5 min at 4°C. Samples were then resuspended in 2 mL of absolute EtOH for 20 min at RT, centrifuged at 7,500 g for 5 min at 4°C, and, after elimination of EtOH by evaporation, stored at −20°C until WB analysis.

### 2.7. Western Blot

The protein pellet was resuspended in distilled water containing 2% sodium dodecylsulfate (SDS) (300 *μ*L/100 mg of initial tissue). Protein concentrations were determined using the Lowry protein assay with bovine serum albumin as the standard. Total proteins (40 *μ*g) were separated by SDS-polyacrylamide gel electrophoresis (SDS-PAGE) using a 10% (w/v) polyacrylamide resolving gel. Internal molecular weight (mw) standards (Kaleidoscope Prestained Standards, Bio-Rad, Hercules, CA, USA) were run in parallel. Two gels were run simultaneously for Coomassie staining and immunoblotting. Proteins for immunoblotting were electrophoretically transferred to a polyvinylidene fluoride membrane (Bio-Rad, Hercules, CA, USA) using the Mini Trans Blot Cell (Bio-Rad, Hercules, CA, USA). Blots were blocked by immersion in 20 mM Tris base and 137 mM sodium chloride (TBS) containing 5% milk powder and 0.1% Tween 20 (TBS-T), for 60 min at room temperature, and incubated overnight at 4°C with a rabbit polyclonal primary antibody against TRPV1 (AbCam, Cambridge, UK), diluted 1 : 1000 in TBS containing 5% milk powder and 0.2% NaN_3_ (Sigma, Milan, Italy). After a TBS-T rinse, blots were incubated for 60 min, at room temperature, with peroxidase-conjugated goat anti-rabbit serum (Sigma-Aldrich, St. Louis, MO, USA), diluted 1 : 10000 in TBS/T. Loading controls were obtained by stripping and immunostaining the membranes as above, using a monoclonal mouse antibody against glyceraldehyde-3-phosphate dehydrogenase (GAPDH) (Chemicon, Temecula, CA, USA), diluted 1 : 1000, as the primary antiserum, and a peroxidase-conjugated goat anti-mouse serum (Chemicon, Temecula, CA, USA), diluted 1 : 5000, as the secondary antiserum. To control for nonspecific staining, the blots were stripped and incubated with the relevant secondary antiserum. After a TBS-T rinse, protein bands were visualized on an autoradiograph (Kodak X-Omat LS, Kodak, Rochester, NY) using the ECL Prime reagents (GE Healthcare, Buckinghamshire, UK). The approximate mw of immunolabeled protein bands was determined by means of Molecular Analyst© Software (Version 1.4, Bio-Rad Hercules, CA, USA) by comparing the position of relevant bands on the autoradiograph film with that of nearby prestained mw standards. Relative optical density (O.D.) obtained by normalizing the optical density of TRPV1-positive bands to that of GAPDH-positive ones was quantified by means of the GS800 Calibrated Densitometer and the Quantity One 1 (Bio-Rad, Hercules, CA, USA) software.

### 2.8. RT-PCR

Total RNA concentration and purity were determined using a nanovolume spectrophotometer (Nanophotometer P 360 Implen GmbH, München, Germany). Total RNA was treated with RNase-free DNase I (RQ1, Promega Corporation, Madison, WI, USA) for 20 min at 37°C. cDNA was then synthesized from the total RNA following a two-step RT-PCR: samples were incubated with Anchored oligo (dT)_23_ primer for 10 min at 70°C then with Enhanced avian RT (Sigma-Aldrich, St. Louis, MO, USA) for 50 min at 42–50°C. RNA was then quantified by spectrophotometry (A260 nm). For PCR amplification, specific primers for TRPV1 and CGRP mRNAs were designed using the http://www.premierbiosoft.com/netprimer/ open source software. Each cDNA was amplified twice. Amplification of the *β*-actin housekeeping gene and mw DNA standards (VWR international, Geldenaaksebaan, Belgium) was run in parallel. Primer base sequences and amplification products are reported in Table 1. Hot start PCR was performed using 20 *μ*L of 1 X Taq DNA Polymerase Master Mix (VWR International, Geldenaaksebaan, Belgium), containing 1.5 mM MgCl_2_, 0.05 U/*μ*L Ampliqon Taq DNA polymerase, 75 mM Tris-HCl, 20 mM (NH_4_)_2_SO_4_, 0.2% Tween 20, 0.2 mM dNTP and a TaqMaster PCR enhancer, 1 *μ*L of 5 *μ*M sense and antisense primers, and 2 *μ*L of cDNA (100 ng/*μ*L), according to the following procedure: 95°C for 3 min, 30–40 cycles 95°C for 30 sec, 54/56°C for 1 min, 72°C for 30 sec, and final elongation at 72°C for 10 min. PCR products were then separated by electrophoresis in a 1.5% agarose gel in TAE buffer (40 mM Tris acetate, 2 mM EDTA) containing GelRed (Biotium, Hayward, CA, USA). cDNA bands were visualized by means of an ultraviolet transilluminator (UVP PhotoDoc-It Imaging System) and digital images of the gel were acquired with a Canon PowerShot A480 camera. The approximate mw of amplification products and relative O.D. was determined on digital images using ImageJ Software (http://rsb.info.nih.gov/ij/), respectively, by comparing the position of relevant bands to that of nearby mw standards and normalizing TRPV1 and CGRP mRNA bands to that of *β*-actin mRNA one.

### 2.9. Immunohistochemistry

A total of 36 DRGs and lumbar spinal cord segments were processed: 16 from the acutely treated group (8 BTZ-treated and 8 controls) and 20 from the chronically treated ones (10 BTZ-treated and 10 controls). Sixteen sciatic nerve segments, 8 from the acutely treated group (4 BTZ-treated and 4 controls) and 8 from the chronically treated ones (4 BTZ-treated and 4 controls), were examined. Cryostat semiconsecutive sections (12 *μ*m thick) were collected on chrome alum-gelatin (USB Corporation, Cleveland, OH, USA) coated slides and processed either by the avidin-biotin-peroxidase complex (ABC) immunohistochemical technique or by immunofluorescence double labeling. Antibodies used as the primary antiserum are reported in Table 2.

For ABC processing, endogenous peroxidase activity was blocked with 0.001% phenylhydrazine in phosphate-buffered saline (PBS) containing 0.2% Triton X-100 (PBS/T) followed by incubation with 20% of either normal goat (NGS) or normal horse serum (Vector, Burlingame, CA, USA) and then incubation with primary antiserum. Biotin-conjugated goat anti-rabbit, donkey anti-goat, and goat anti-guinea-pig serum (Vector, Burlingame, CA, USA), diluted 1 : 400, were used as secondary antiserum. The reaction product was revealed by the ABC complex (BioSpa Division, Milan, Italy), diluted 1 : 250, followed by incubation with a solution of 0.1 M PB, pH 7.3, containing 0.05% 3,3′-diaminobenzidine (Sigma, Milan, Italy), 0.04% nickel ammonium sulfate, and 0.01% hydrogen peroxide. Incubations with primary antiserum were carried out overnight at 4°C. Incubation with secondary antiserum and ABC lasted 60 min and was performed at RT. All antisera and the ABC were diluted in PBS/T.

For double labeling, after a preincubation with 20% NGS, sections were double stained for TRPV1/CGRP and TRPV1/SP by means of subsequent incubations with primary antiserum, diluted as reported in Table 2. Sections were then incubated with Alexa Fluor 488- or Alexa Fluor 594-conjugated secondary antibodies (Invitrogen, Eugene, OR, USA), diluted 1 : 500 and 1 : 600, respectively. Control immunostaining was obtained by substituting the primary antiserum with normal serum. Slides were examined with an Olympus BX61 microscope, equipped with epifluorescence illumination, and digital images were captured with a Leica DF 450C camera.

### 2.10. Morphometry

Morphometric analysis, carried out by the same examiner blinded to animals' treatment, was performed on DRG neuronal cell profiles in digital images captured with a 20x objective magnification from 10 to 12 animals out of the acutely and chronically treated groups, respectively. The tissue sections were separated by at least 108 *μ*m and only cells that obviously showed the nuclear profile were considered. Neuronal mean diameters were automatically measured by ImageProPlus software. Statistical parameters (mean, minimum, maximum, and SD) and histograms of the neuron sizes, processed as a pool, were obtained by Statistica 6 software. The percentage of DRG immunostained perikarya, processed as a pool, was calculated as the ratio of the total number of labeled cells found in four to eight sections to the total number of cells found in the same sections after a modified Mayer's hematoxylin (certified hematoxylin (1.0 g/L), sodium iodate (0.2 g/L), aluminium ammonium sulfate*·*12 H_2_0 (50 g/L), chloral hydrate (50 g/L), and citric acid (1 g/L)) counterstaining.

### 2.11. Image Densitometry

For the quantitative evaluation of SP, CGRP, and TRPV1 immunohistochemical labeling in the dorsal horn of the spinal cord, 6 representative 10x magnification microscopic fields for each marker (three BTZ-treated animals and three controls) were blindly analyzed with ImageJ (http://rsb.info.nih.gov/ij/). Mean gray values from negative controls were subtracted from the gray values of the immunostained sections to exclude background staining. Histograms were obtained by calculating the ratio of BTZ-treated values to control values.

### 2.12. Statistical Analysis

The differences in body weight, nerve conduction velocity, and behavioral data were statistically evaluated using unpaired Student's *t*-test (significance level set at *P* < 0.05). Paired Student's *t*-test was used for comparing differences between percent frequencies of DRG labeled neurons, relative frequencies of neuronal size groups, and grey levels. One-way ANOVA and Fisher's test for post hoc analyses were applied to evaluate statistical differences among groups in the western blot and RT-PCR analysis.

## 3. Results

### 3.1. General Toxicity

**Figure 1:**
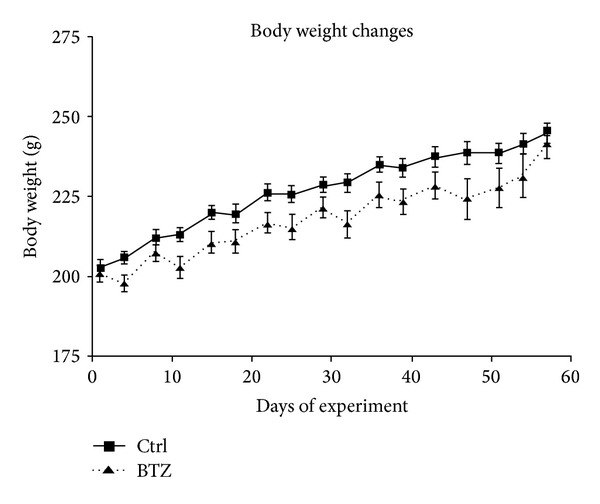
Body weight changes throughout the study. No obvious differences in weight gain versus control (ctrl) are evident during chronic BTZ-treatment (mean values ± SD).

No acute distress was observed in the rats sacrificed 1 hour after BTZ administration. In the chronic experiment, 8 animals treated with BTZ died within the first 4 weeks of administrations, while treatment was well tolerated by the surviving animals. During the first week of treatment some of the animals showed mild signs of discomfort such as piloerection, hypokinesia, and chromodacryorrhea. After a slight and not statistically significant decrease in body weight in the first 10 days of administration, the BTZ-treated animals' body weight gain assumed the same trend of the control rats. At the end of drug treatment, no significant difference was observed in the body weight of control and BTZ-treated animals (Figure 1).

### 3.2. Neurotoxicity and Behavioral Measures

**Figure 2:**
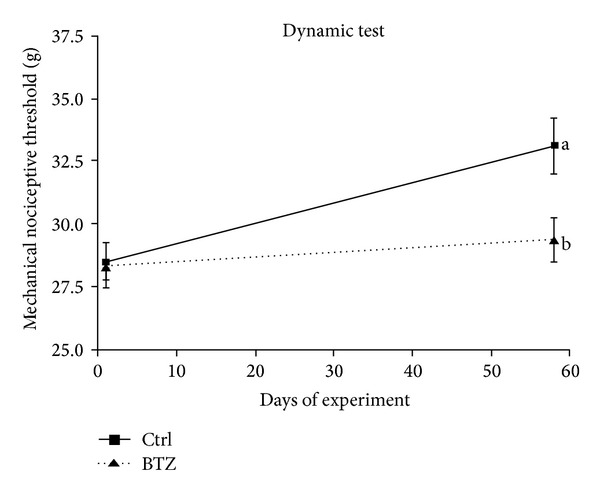
Bortezomib-induced changes in Dynamic Aesthesiometer test results. The mechanical threshold was measured before starting the pharmacological treatment (baseline value) and after 8 weeks of chronic treatment (mean values ± SD, *n* = 12 in each group during the treatment period).

As previously observed [28, 29], BTZ induced functional and structural damage in the peripheral nervous system after chronic administration. Since the acute model animals are sacrificed one hour after BTZ administration, neurophysiological measurements were performed at the end of the 8-week treatment period in the chronic model and confirmed that BTZ induced a statistically significant reduction of the caudal nerve conduction velocity (mean controls: 31.2 ± 5.4 m/sec; mean BTZ-treated: 18 ± 6.6 m/sec; *P* < 0.001, data not shown). At the end of the 8-week treatment period BTZ also induced a statistically significant reduction of the mechanical nociceptive threshold (mean controls: 32.6 ± 2.1; mean BTZ-treated: 27.4 ± 2.6;  *P* < 0.01; Figure 2). By contrast, and in complete agreement with previous studies [29, 30], no difference in the thermal withdrawal latency was observed between BTZ-treated and untreated groups (mean controls: 12.0 ± 0.2; mean BTZ-treated: 9.5 ± 0.2; data not shown).

Histopathological analysis revealed that administration of BTZ did not induce morphological alterations in the specimens obtained from acutely treated rats. In chronically treated animals, the expected BTZ-induced mild axonopathy previously reported [28, 29] was present in the sciatic nerve, affecting mostly the small myelinated and unmyelinated fibers. Similarly, clear cytoplasmic vacuolization in some DRG satellite cells was detected, while no obvious changes were observed in the dorsal horn of the spinal cord (data not shown).

### 3.3. TRPV1, CGRP, and SP Expression and Localization

BTZ-treatment variously affected TRPV1, CGRP, and SP expression in the DRGs, spinal cord, and sciatic nerve.

#### 3.3.1. Western Blot

**Figure 3:**
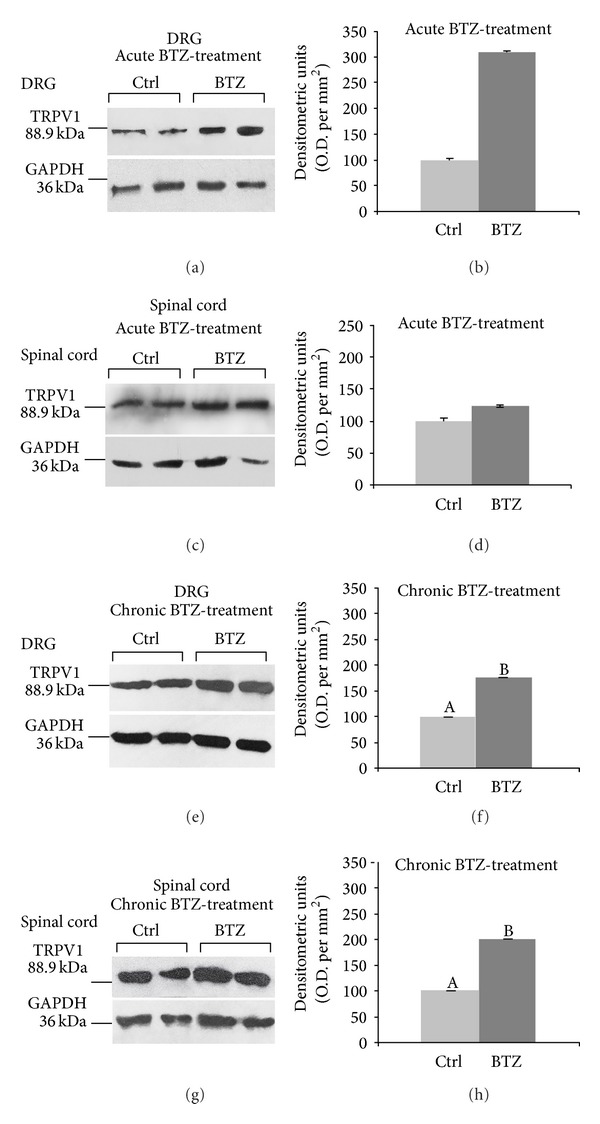
Western blot analysis of TRPV1 in DRG ((a) and (e)) and spinal cord ((c) and (g)) of acutely and chronically BTZ-treated rats. (b), (d), (f), and (h) Relative levels of TRPV1 expression in DRG and spinal cord with densitometric analysis of the grey levels expressed as a percentage of the optical density (O.D.) ratio of the TRPV1-positive bands to the GAPDH-positive ones. Ctrl: control rats. Error bars represent standard deviation. Letters “A” and “B” denote significant differences (*P* < 0.05).

The antibody against TRPV1 labeled a single protein band at the expected mw of 88.9 kDa (Figure 3) [49] in DRGs and spinal cord tissue homogenates. In both acutely and chronically BTZ-treated rats, TRPV1 protein levels were higher than in control animals. After acute treatment, relative optical density (O.D.) of TRPV1 protein bands increased threefold in DRG homogenates (Figures 3(a) and 3(b)) (*P* < 0.05) whereas TRPV1 protein relative levels did not change in the spinal cord (Figures 3(c) and 3(d)). Chronic BTZ-treatment produced a statistically significant increase in the relative O.D. of the TRPV1 protein band in both DRGs (Figures 3(e) and 3(f)) and spinal cord (Figures 3(g) and 3(h)), amounting to 75% (*P* < 0.05) and 100% (*P* < 0.05), respectively, in BTZ-treated versus control animals.

#### 3.3.2. RT-PCR

**Figure 4:**
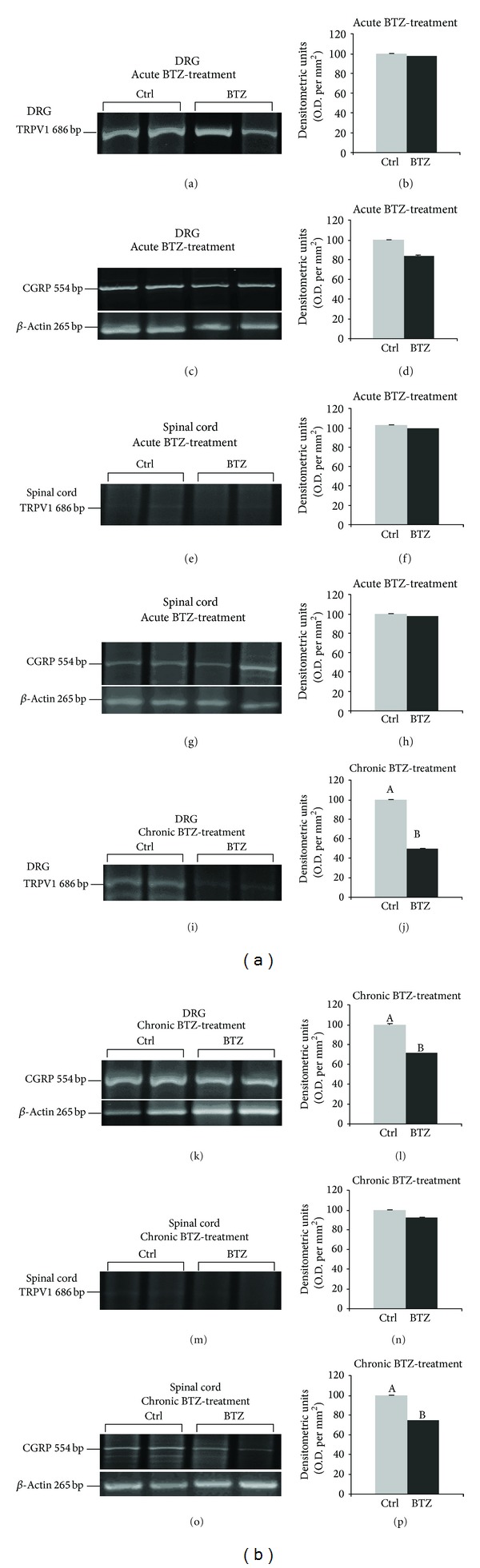
RT-PCR analysis of TRPV1 and CGRP mRNAs in DRG ((a), (c), (i), and (k)) and spinal cord ((e), (g), (m), and (o)) of acutely and chronically BTZ-treated rats. (b), (d), (f), (h), (j), (l), (n), and (p) Relative levels of TRPV1 and CGRP expression with densitometric analysis of the grey levels expressed as a percentage of the optical density (O.D.) ratio of the TRPV1- and CGRP-positive bands to the relevant GAPDH-positive ones. Ctrl: control rats. Error bars represent standard deviation. Letters “A” and “B” denote significant differences between relative mRNA levels in ctrl versus BTZ-treated rats ((b) *P* < 0.001; (d) and (h) *P* < 0.05).

After acute BTZ-treatment, no significant changes in the relative O.D. of TRPV1 mRNA bands occurred in the DRGs (Figures 4(a) and 4(b)) and spinal cord (Figures 4(e) and 4(f)). In addition, relative O.D. of CGRP mRNA bands was unaltered in the DRGs (Figures 4(c) and 4(d)) and spinal cord (Figures 4(g) and 4(h)). After chronic BTZ-treatment, a significant decrease of the relative O.D. of TRPV1 mRNA band occurred in the DRGs (−50%, *P* < 0.001; Figures 4(i) and 4(j)), but not in the spinal cord (Figures 4(m) and 4(n)), while a significant decrease of the relative O.D. of CGRP mRNA band occurred in the DRGs (−28%, *P* < 0.05; Figures 4(k) and 4(l)) as well as in the spinal cord (−26%; *P* < 0.05; Figures 4(o) and 4(p)).

#### 3.3.3. Immunohistochemistry

**Figure 5:**
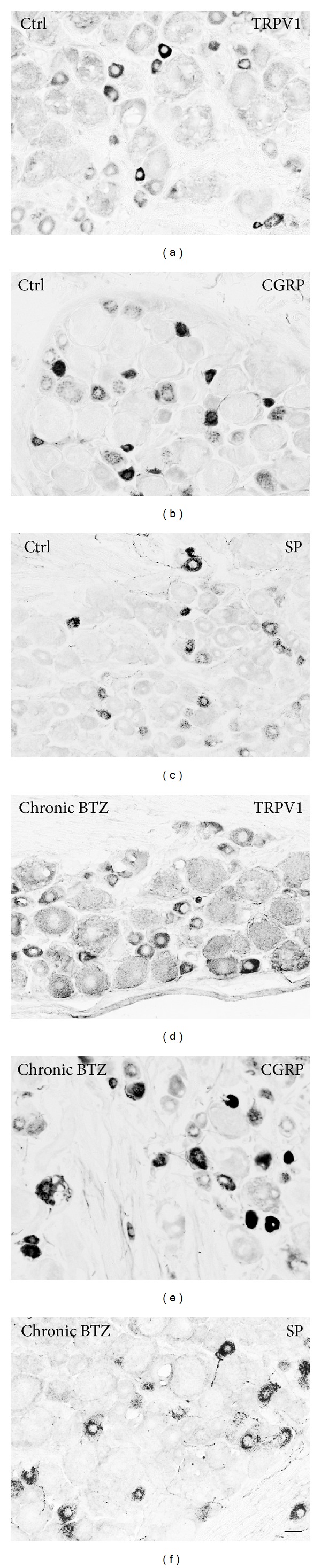
Immunoreactivity to TRPV1 ((a) and (d)), CGRP ((b) and (d)), and SP ((e) in representative sections of lumbar DRG from control (ctrl) ((a), (c), and chronically BTZ-treated rats ((d), (e), and (f)). Scale bar = 25 *μ*m.

**Figure 6:**
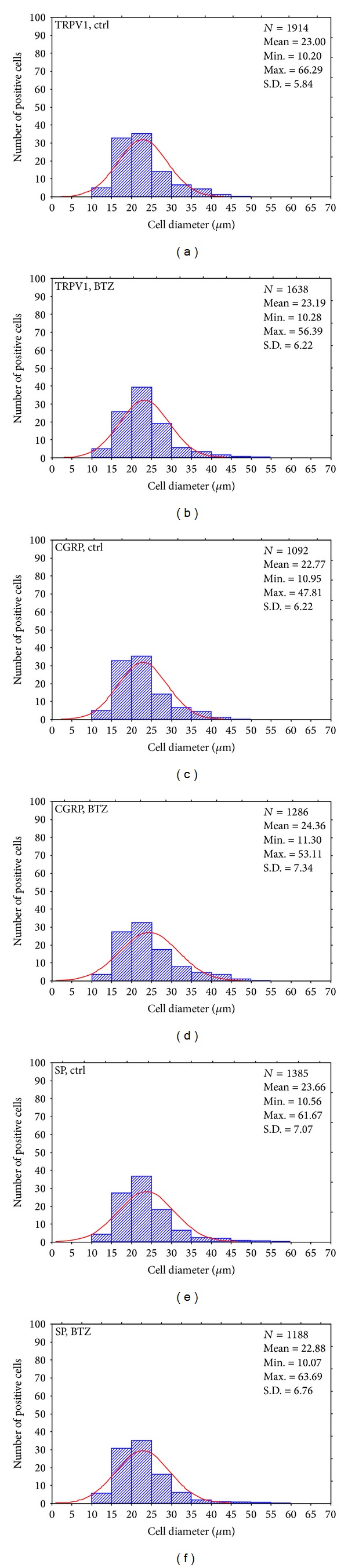
Size frequency histogram of TRPV1-, CGRP-, and SP-LI DRG neurons from control (ctrl) and chronically BTZ-treated rats. Cells present in at least 6 sections were measured. *x*-axis values represent the mean cell diameters expressed in *μ*m; *y*-axis reports values of relative percent frequency. Curve superimposed on the histogram represents the theoretical normal distribution. *N*: total number of sized positive neurons; SD: standard deviation.

*DRGs*. TRPV1-, CGRP-, and SP-like immunoreactive (LI) neurons were fairly heterogeneous in both the density of reaction product and the cell size after acute (Supplementary Figures  1 and  2 in Supplementary Material available online at http://dx.doi.org/10.1155/2014/180428) and chronic (Figures 5 and 6) BTZ-treatment.

The immunolabeling had a granular appearance and it was distributed throughout the cytoplasm. Uneven positive staining, suggestive of a discrete localization in the Golgi apparatus and Nissl substance, could be seen in a number of immunostained neurons. TRPV1-, CGRP-, and SP-LI nerve fibers were also present between neuronal cell bodies and in nerve bundles. After acute treatment (Supplementary Figure  1 and Table 3), the proportion of DRG labeled neurons increased by about 3% (*P* < 0.005) for TRPV1 and about 10% for CGRP (*P* < 0.001), whereas it did not change for SP. In some specimens, peripheral neuronal SP-like immunoreactivity suggestive of labeled satellite cells was observed (Supplementary Figure  1(g)). After chronic BTZ-treatment (Figure 4 and Table 3), the proportion of TRPV1-LI neurons significantly increased by about 11% (*P* < 0.001) and that of CGRP-LI neurons by about 5% (*P* < 0.001), whereas it did not change for the SP-LI neuronal subpopulation.

The mean cell diameter of TRPV1-, CGRP-, and SP-LI DRG neurons ranged from 8 to 55 *μ*m in acutely treated rats and from 10 to 65 *μ*m in chronically treated animals. Size frequency histograms of labeled neurons in acutely and chronically treated rat DRGs are shown in Supplementary Figure 2 and Figure 5, respectively. The majority of measured neurons were in the range of small- (diameter < 25 *μ*m) and medium- (25–35 *μ*m) sized cells, with a mean cell diameter between 15 *μ*m and 30 *μ*m. Moreover, for all markers, a statistically significant rearrangement in the size distribution of positive neurons was observed in both acutely (Supplementary Figure  2) and chronically treated rats (Figure 5), with differential changes involving the relative frequency of subcategories of small- and medium-sized immunoreactive neurons. In particular, after acute treatment, the TRPV1-, CGRP-, and SP-LI small neurons with a mean diameter comprised between 5 and 20 *μ*m decreased, whereas those with a mean diameter > 25 *μ*m increased in number. After chronic treatment, TRPV1-LI small (and in particular those between 15 and 25 *μ*m) and medium-large neurons (and in particular those between 35 and 60 *μ*m) slightly increased, whereas the number of stained neurons falling in the range of 25–30 *μ*m decreased. CGRP-LI neurons with a mean cell diameter of 10–25 *μ*m decreased, while those between 25 and 55 *μ*m increased. A slight but nonsignificant increase occurred in the SP-LI neurons with a mean cell diameter of 10–20 *μ*m (*P* = 0.058).

**Figure 7:**
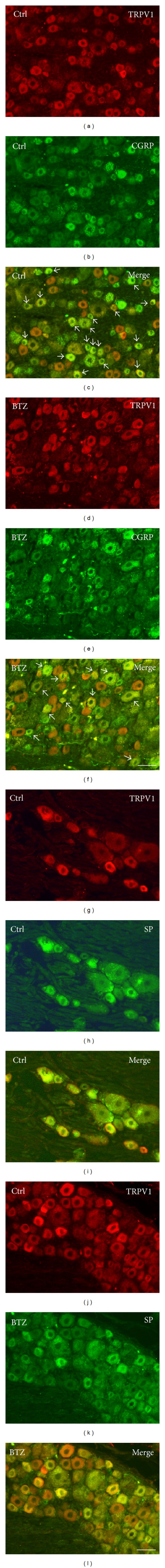
Double labeling immunofluorescence for TRPV1/CGRP ((a–c) and (d–f)) and TRPV1/SP ((g–i) and (j–l)) in DRG neurons from control (ctrl) ((a), (d), (g), and (j)) and chronically BTZ-treated rats ((b), (e), (h), and (k)). (c), (f), (i), and (l) represent the composite images obtained by overlay of (a)-(b), (d)-(e), (g)-(h), and (j)-(k), respectively. Scale bar = 25 *μ*m.

Analysis of double immunofluorescence-stained tissue for TRPV1 and either neuropeptide revealed that the neuronal coexpression of TRPV1/SP and TRPV1/CGRP is partial, occurring in approximately 45% of TRPV1-LI neurons in control animals. An estimation of changes in TRPV1/either neuropeptide coexpressing neurons suggested that, in the acutely treated group (Supplementary Figure  3), the TRPV1/CGRP subpopulation did not show any statistically significant change, whereas the TRPV1/SP one decreased significantly (−12%, *P* < 0.05; Table 4). In the chronically treated group (Figure 7), both TRPV1/CGRP- (−7%; *P* < 0.05) and TRPV1/SP-coexpressing neurons (−8%, *P* < 0.005) showed a statistically significant decrease (Table 4). 

**Figure 8:**
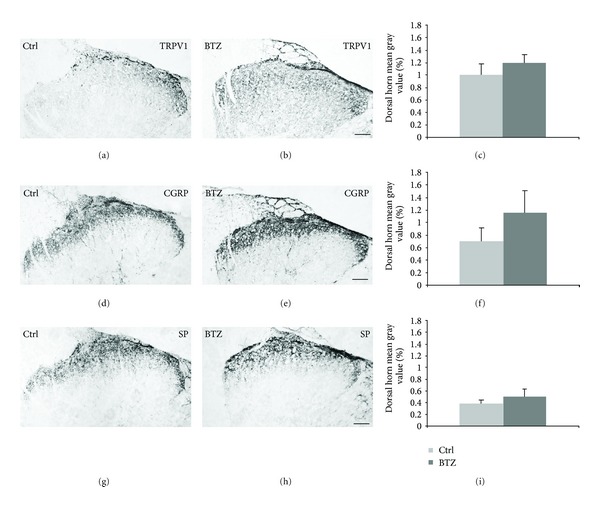
Immunoreactivity to TRPV1 ((a), (b), and (c)), CGRP ((d), (e), and (f)), and SP ((g), (h), and (i)) in representative sections of lumbar spinal cord dorsal horn from control (ctrl) ((a), (d), and (g)) and chronically BTZ-treated rats ((b), (e), and (h)). Scale bar = 100 *μ*m.

**Figure 9:**
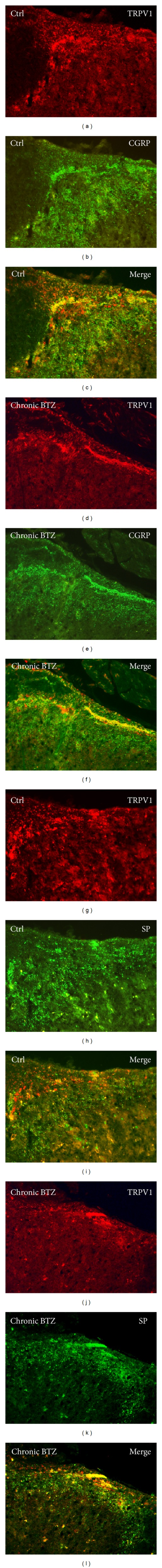
Double labeling immunofluorescence for TRPV1/CGRP ((a–c) and (d–f)) and TRPV1/SP ((g–i) and (j–l)) in lumbar spinal cord dorsal horn from control (ctrl) ((a–c) and (g–i)) and chronically BTZ-treated rats ((d–f) and (j–l)). (c), (f), (i), and (l) represent the composite images obtained by overlay of (a)-(b), (d)-(e), (g)-(h), and (j)-(k), respectively. Scale bar = 25 *μ*m.

*Spinal Cord*. The majority of immunoreactivity to TRPV1, CGRP, and SP was found in the dorsal horn (Supplementary Figure  4; Figure 8). TRPV1-LI structures were prominent in Lissauer's tract and lamina I and occurred with a lighter labeling in inner lamina II. CGRP- and SP-LI structures showed a wider distribution, being present in Lissauer's tract, laminae I-III, and lamina V. Image densitometric analysis of dorsal horn labeling showed a statistically significant increase in CGRP immunostaining in the acutely treated animals (*P* < 0.05) and a trend to increase in chronically treated ones (*P* = 0.057) (Supplementary Figure  4; Figure 8). No changes were found in the ventral horn, where CGRP immunolabeled motoneurons were detectable. Double immunofluorescence for TRPV1 and either neuropeptide showed that the markers were mainly codistributed in the spinal cord. However colocalization was scarce with respect to the coexpression detected in DRG neurons, being limited to nerve bundles in Lissauer's tract and in lamina I (Figure 9). No evident differences in the degree of codistribution/colocalization could be appreciated in BTZ-treated compared to control rats. 

**Figure 10:**
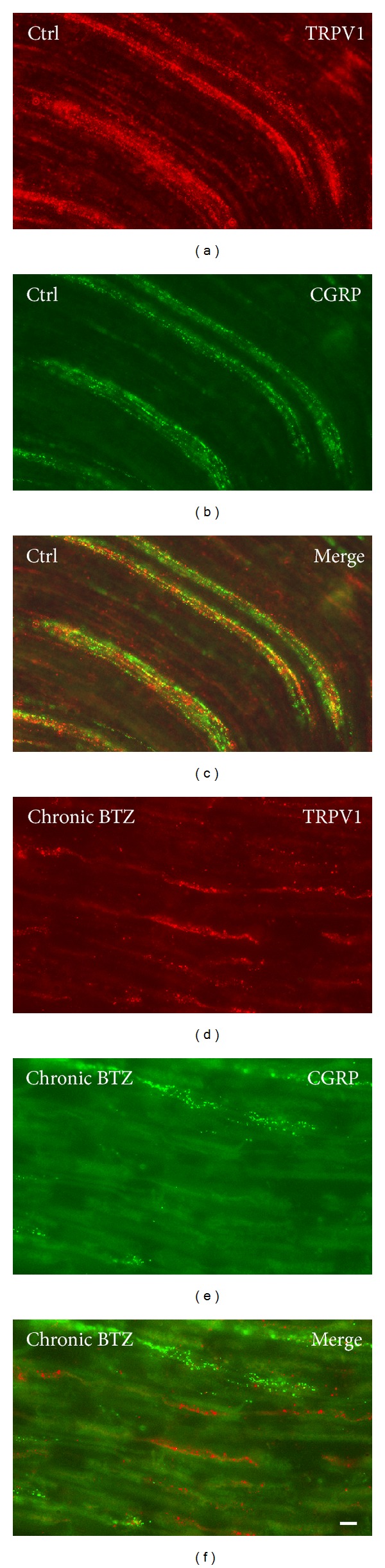
Double labeling immunofluorescence for TRPV1/CGRP in sciatic nerve from control (ctrl) (a–c) and chronically BTZ-treated rats (d–f). (c) and (f) represent the composite images obtained by overlay of (a)-(b) and (d)-(e), respectively. Scale bar = 10 *μ*m.

*Sciatic Nerve*. TRPV1-, CGRP, and SP-LI nerve fibers were easily detectable in longitudinal sections of sciatic nerves. No apparent differences between treated and control animals were found. Analysis of colocalization performed on double immunofluorescence preparations for TRPV1 and CGRP revealed that, in both acutely (Supplementary Figure  5) and chronically treated animals (Figure 10), TRPV1-like immunoreactivissty occurred mainly in fibers that did not show CGRP labeling. Due to the paucity of double-labeled structures, no quantitative evaluation was performed.

## 4. Discussion 

The aim of this study was to provide a neurochemical characterization of the events likely involved in the onset and persistence of nociceptive symptoms caused by BTZ-induced neurotoxicity occurring in DRGs and peripheral nerve fibers. Although the BTZ schedule used in this study differs from that used in clinical practice with respect to the duration of administration and dosage, calculations to translate drug doses from animal to human studies [50] indicate that our model of BTZ-induced neurotoxicity is highly relevant since it shares common neurophysiological, behavioral, and pathological features with the sensory neuropathy described in humans [17, 27]. Moreover, like the effect on patients, the selected dose-schedule combination induces inhibition of proteasome activity at more than 80% [51] and neurological impairment persists during the follow-up period [28, 29, 51]. Our results demonstrate that the chronic BTZ-treatment affects the morphological and functional integrity of peripheral small myelinated and unmyelinated nerve fibers and DRG satellite cells and modifies nociceptive behavior. Neurophysiological and neuropathological assessments were consistent with measures at baseline and end of treatment time points previously observed [28, 29]. The chronic model of BTZ-induced PN is associated with the onset of mechanical allodynia as assessed after the 8-week treatment. Interestingly, data concerning the weekly outcome of BTZ-treatment showed a significant reduction in withdrawal latency after 4 weeks of treatment; however, at the later time point of 8 weeks (end of treatment period) and the follow-up 4-week period, the difference was no longer significant [28].

The present study provides novel evidence that BTZ-induced nocifensive behavior is accompanied by increased TRPV1 protein levels in DRGs and spinal cord and downregulated levels of TRPV1 mRNA and CGRP mRNA. Though details about neuronal mechanisms underlying the protein/mRNA reciprocal changes remain to be elucidated, it has been recently shown that proteasomal degradation is implicated in TRPV1 catabolism by regulating the balance between neuronal receptor synthesis and degradation [52]. In particular, TRPV1 is prone to ubiquitination and susceptible to proteasomal degradation, especially when its plasma membrane trafficking is blocked [53]. In both rat [40] and mouse models of BTZ-induced PN [53, 54], the occurrence of axonal degeneration and Schwann cell endosomal membrane dilations likely accounts for impaired protein membrane trafficking that, given the enduring BTZ-induced proteasome inhibition, might cause TRPV1 accumulation in the cytoplasm. Support for this hypothesis derives also from mitochondrial toxicity and endoplasmic reticulum stress in Schwann cells following BTZ-treatment and leading to maladaptive responses such as demyelination and macrophage recruitment [53]. As for the protein/mRNA differential regulation [55], the direct action of BTZ on the ubiquitin-proteasome system and the possible inhibition of NF-*κ*B activation may account for it. In fact, the effects of proteasome blockade are cytoplasmic accumulation of ubiquitin-protein conjugates, reduction of transcriptional activity, and nuclear retention of poly(A)RNAs [14–17, 26]. It is also conceivable that the increase in protein expression acts as a retrograde signal to modulate transcription. Yet, NF-*κ*B proteasome-independent activation pathways have also been reported [56, 57] and, depending on the targeted cell type, BTZ may activate rather than inhibit the NF-*κ*B canonic pathway [58]. Plasticity of TRPV1 expression in chronic pain conditions is considered to represent one of the mechanisms involved in hyperalgesia [59]. Regarding the specific time profile and turnover of TRPV1 protein after the acute BTZ delivery, we were not able to find literature data with which to compare our results, since experimental studies on the effect of a BTZ single dose, both in rats [26, 51] and mice [60], were not designed to investigate the immediate neurotoxicity of its administration. However, pharmacokinetics of BTZ are well known as the drug has been shown to be rapidly distributed into tissues after administration of a single dose, with an initial plasma distribution half-life of less than 10 minutes. Maximum proteasome inhibition occurs within 1 hour and recovers close to baseline within 72 to 96 hours after administration [61]. Recently, in the same rat model of BTZ-induced PN as ours and with the same dose used in this study, Meregalli et al. [51] found that a maximal time-dependent proteasome inhibition can be observed in peripheral blood mononuclear cells and sciatic nerve specimens by 1 hour from a single dose BTZ delivery, thus supporting the possibility of a quick cytoplasmic accumulation of TRPV1 protein, as also suggested by the low but significant increase of the proportion of TRPV1-LI neurons in the DRG (present data).

BTZ-treatment also induced an augmented immunoreactivity to TRPV1 and CGRP in the DRG neurons and spinal dorsal horn and caused a decrease of TRPV1/neuropeptide colocalization in DRG neurons. Although the functional characterization of TRPV1-labeled neurons goes beyond the aim of this study, available experimental studies on the systemic administration of different TRPV1 antagonists demonstrate a reduction of mechanical allodynia and hyperalgesia in animal models of neuropathic, inflammatory, and postoperative pain [33–36, 62, 63], suggesting a role of TRPV1 in the modulation of mechanical hyperalgesia.

Recent experimental studies have further contributed to the understanding of the pathogenesis of BTZ-induced peripheral neurotoxicity [26, 28, 29, 54, 64, 65]. Although patterns differ by species and duration of treatment, peripheral nerve axons and DRGs have been found to be selectively vulnerable to changes secondary to BTZ administration [28, 29, 40, 54], whereas no reports on central nervous system damage are available due to the inability of BTZ to cross the blood-brain barrier [66]. However, despite evidence of BTZ-induced neuronal dysfunction in cultured DRG neurons [26], tissue analysis at the light and electron microscope level has failed to demonstrate severe abnormalities in DRG neuronal perikarya or in their centripetal projections to the spinal dorsal horn [28, 29].

In agreement with the clinical features seen in patients treated with BTZ who develop painful peripheral neuropathy after the first cycle of treatment [20], we demonstrate that a single dose of the drug is sufficient to trigger changes in the relative amounts of both protein and mRNA levels. The neuronal response to BTZ neurotoxicity not only involves the DRGs but also extends to the spinal cord dorsal horn, where neurochemical alterations parallel those observed in the periphery. In particular, following BTZ-treatment, TRPV1 protein level and density of TRPV1- and CGRP-immunolabeling are increased, while the relative amount of TRPV1 mRNA and CGRP mRNA is decreased. Importantly, this response is larger in the chronically BTZ-treated animals.

The basal expression of TRPV1 mRNA in the spinal cord is in keeping with previous RT-PCR studies [67, 68] and with the recent demonstration that TRPV1 expression occurs in GABAergic interneurons of the rat dorsal horn, where it mediates neuropathic mechanical allodynia and disinhibits nociceptive circuits in the spinal cord [69]. Interestingly, TRPV1 expression by glial cells has also been described [70] and TRPV1 has been reported to be involved in activating spinal glia in mice with nociceptive and pathological pain [71]. Moreover, since bidirectional axonal transport of TRPV1 mRNA along primary afferents has been demonstrated in some pathological conditions such as experimental acute inflammation [72], it cannot be excluded that part of the TRPV1 mRNA detected in our specimens has a primary afferent origin. If this was the case, the reduction of TRPV1 mRNA secondary to BTZ administration might be explained by the reduction of its production and hence centripetal transport, by DRG neurons.

Consistent with changes in TRPV1 transcript and protein levels, CGRP mRNA also showed a decrease that may be correlated to the increase in density of CGRP-immunoreactive structures in the BTZ-treated versus control rats.

While experimental evidence regarding the effects of BTZ on the neurochemical phenotype is limited to data on CGRP and TRP receptors in mouse models, after acute [60] and chronic administration [54, 60], several studies of sensory neuropeptides [73–75] and TRP receptors in the spinal cord and DRG [76–79] following treatment with different classes of antineoplastic drugs are available in rodents. It has been demonstrated for instance that platinum-based compounds, taxanes, and vinca alkaloids modulate the expression of TRPV1, as well as other TRP receptors, in DRG neurons* in vitro* and* in vivo *[77–79]. Cisplatin and oxaliplatin affect the expression of TRPV1 and TRPA1 mRNA by causing a 3-fold mRNA increase as early as 6 hours after treatment in cultured DRG neurons [77]. Similarly, trigeminal ganglia (TG) of cisplatin-treated mice had significant increases in TRPV1 mRNA expression [77]. However, mRNA changes are not mirrored by immunohistochemistry studies, showing that no change occurred in the proportion of the TRPV1 immunopositive TG neurons in cisplatin- and oxaliplatin-treated mice compared to naïve animals [77]. Similarly, TRP receptors have been shown to have a role in the pathogenesis of paclitaxel-associated pain symptoms that were related to increased TRPV1 mRNA and protein expression in rat DRG neurons [79]. A single injection of paclitaxel is sufficient to increase the expression of TRPV1 mRNA and protein in rat DRG (as seen in both homogenates and tissue sections by RT-PCR, in situ hybridization, and immunochemistry) and to enhance TRPV1 expression in the paw skin at day 14 after treatment [79]. By contrast, the development of sensory disorders following vincristine treatment has been related to changes in the expression of TRPV4 [76]. To our knowledge this is the first study reporting that BTZ-treatment in a rat model causes a general increase in the percent frequency of TRPV1- and CGRP-LI DRG neurons after both acute and chronic administration. Our immunohistochemical data are in keeping with those on CGRP-LI neuronal frequency in mouse DRGs after chronic BTZ-treatment [54]. The increase in TRPV1- and CGRP-LI in the DRGs occurs with a parallel increase in the spinal cord dorsal horn. Interestingly, increased immunoreactivity for TRPV1 [62, 79, 80] and CGRP [56, 81] has been previously shown in uninjured primary afferent neuronal perikarya in different models of nerve lesion. Moreover, CGRP expression may increase in several pathological conditions, including partial nerve injury, where pain hypersensitivity occurs [82–86].

In our model, BTZ administration induced a rearrangement in the relative size frequency of TRPV1-, CGRP-, and SP-LI neuronal subgroups, suggesting the possibility of a drug-induced neuronal phenotype switch that may go with the onset of BTZ-induced mechanical hyperalgesia. These observations are in agreement with those obtained in the mouse model of BTZ neurotoxicity where medium-sized neurons slightly decrease, small neurons increase, and the relative number of large CGRP-LI neurons increases [54]. Taken together, these data suggest that the phenotypic switch secondary to BTZ-treatment may partly represent the neuronal response to treatment, as also suggested by Bruna et al. [54].

Overall, the colocalization data support that a portion of the TRPV1-LI DRG neurons are neuropeptidergic. The analysis of changes in neuronal colocalization of TRPV1 with neuropeptides provides a morphological basis to suggest a role for TRPV1 in the release of these molecules and thus in nociceptor sensitization and supports the possible implication of TRPV1-, CGRP-, and SP-positive neurons in the persistence of painful neuropathic symptoms [37]. The fact that the TRPV1/neuropeptide colocalization seen in the DRGs did not parallel that observed in both central and peripheral nerve processes of primary afferent neurons is in keeping with previous observations by Guo [87], who suggested alternative mechanisms of neuropeptide release in response to capsaicin application. Future studies will examine how these neurochemical changes affect neurophysiological function in DRG neurons and the spinal dorsal horn.

## 5. Conclusions

Our data support the notion that BTZ-treatment selectively affects primary sensory neurons that are likely involved in the processing of painful sensory stimuli. In DRG neurons and the spinal cord, the BTZ-induced neurochemical changes appear after a single dose of the drug and are enhanced after chronic treatment. Taken together, these findings increase our understanding of the nociceptive symptoms associated with BTZ-induced sensory neuropathy and may contribute to the development of new targeted therapies.

## Supplementary Material

Supplementary Figures 1 and 2, respectively, show TRPV1-, CGRP-, and SP-LI DRG neurons in control and acutely BTZ-treated rats and their relevant size frequency histograms.
Supplementary Figure 3: shows double staining immunofluorescence for TRPV1 and either CGRP or SP in the DRG of control and acutely BTZ-treated rats.
Supplementary Figure 4: shows immunoreactivity to TRPV1, CGRP, SP and relevant densitometry of immunostained sections in the dorsal horn of control and acutely treated rats.
Supplementary Figure 5: shows the outcome of immunostaining for TRPV1, CGRP, and SP in sciatic nerve sections of control and acutely treated rats.

## Figures and Tables

**Table 1:** Primer sequences for RT-PCR.

mRNA	Primer sequences	Amplification product
TRPV1	Sense: 5′-gcactcctccctttatgac-3′ Antisense: 5′-gccgatggtgaacttgaac-3′	686 bp fragment (NCBI Ref. sequence: NM_031982.1)

CGRP	Sense: 5′-ctctcagcagcatgtgggt-3′ Antisense: 5′-taactcatttatacttggtttca-3′	554 bp fragment (NCBI Ref. sequence: NM_138513.1)

*β*-Actin	Sense: 5′-ccagagcaagagaggcatc-3′ Antisense: 5′-gtccctgtat gcctctggt-3′	265 bp fragment (NCBI Ref. sequence: NM_031144.2)

**Table 2:** Antibodies used for immunohistochemistry.

Antigen	Immunogen	Manufacturer, species, and type	Dilution used
TRPV1	Synthetic peptide, RASLDSEESESPPQENSC corresponding to aa 4–21 of rat TRPV1	Neuromics Rabbit Polyclonal	1 : 6000 (ABC), 1 : 1000 (IF),
	Synthetic peptide, CGSLKPEDAEVFKDSMVPGEK corresponding to C-terminal residues 816–838 of rat VR1	AbCam Rabbit Polyclonal	1 : 600 (ABC), 1 : 100 (IF), 1 : 1000 (WB)
	Epitope mapping near N-terminus of VR1 of rat origin	SantaCruz Biotechnology Goat Polyclonal	1 : 800 (ABC), 1 : 100 (IF)

CGRP	Synthetic CGRP from rat	Chemicon Int. Rabbit Polyclonal	1 : 1200 (ABC), 1 : 600 (IF)
	Synthetic rat alpha-CGRP conjugated to bovine serum albumin using glutaraldehyde	Enzo Life Sciences Rabbit Polyclonal	1 : 5000 (ABC), 1 : 2000 (IF)
	Rat alpha-CGRP peptide	AbCam Mouse Monoclonal	1 : 500 (ABC), 1 : 100 (IF)

SP	Synthetic peptide: CRPKPQQFFGLM, corresponding to amino acids 1–11 of rat substance P	AbCam Guinea pig Polyclonal	1 : 1500 (ABC), 1 : 500 (IF)

GAPDH	GAPDH from rabbit muscle	Millipore Chemicon	1 : 1000 (WB)

**Table 3:** Percentage (±confidence limits) of TRPV1-, CGRP- and SP-LI DRG neurons in BTZ-treated rats.

Acute BTZ-treatment		
	Ctrl	BTZ
TRPV1	26.86 ± 0.01%	29.55 ± 0.01%
CGRP	25.32 ± 0.01%	34.89 ± 0.01%
SP	15.86 ± 0.04%	14.73 ± 0.03%

Chronic BTZ-treatment		
	Ctrl	BTZ

TRPV1	28.65 ± 0.02%	39.93 ± 0.03%
CGRP	28.59 ± 0.02%	34.87 ± 0.02%
SP	20.60 ± 0.01%	19.85 ± 0.01%

**Table 4:** Percentage (±confidence limits) of TRPV1/neuropeptide DRG neurons in BTZ-treated rats.

Acute BTZ-treatment		
	Ctrl	BTZ
TRPV1/CGRP	43.68 ± 0.06% (58 colocalized/87 TRPV1)	49.43 ± 0.11% (43 colocalized/87 TRPV1)
CGRP/TRPV1	39.58 ± 0.10% (58 colocalized/96 CGRP)	41.35 ± 0.01% (38 colocalized/104 CGRP)
TRPV1/SP	43.53 ± 0.11% (37 colocalized/85 TRPV1)	32.33 ± 0.06 %* (86 colocalized/266 TRPV1)
SP/TRPV1	35.92 ± 0.1% (37 colocalized/103 SP)	44.10 ± 0.07%^§^ (86 colocalized/195 SP)

Chronic BTZ-treatment		
	Ctrl	BTZ

TRPV1/CGRP	43.86 ± 0.092% (50 colocalized/114 TRPV1)	36.84 ± 0.11% (28 colocalized/76 TRPV1)
CGRP/TRPV1	36.76 ± 0.003% (50 colocalized/136 CGRP)	27.72 ± 0.09%^§§^ (28 colocalized/101 CGRP)
TRPV1/SP	43.33 ± 0.1% (39 colocalized/90 TRPV1)	24.81 ± 0.07%** (33 colocalized/133 TRPV1)
SP/TRPV1	50.00 ± 0.006% (39 colocalized/78 SP)	61.11 ± 0.13% (33 colocalized/54 SP)

*P* value of ctrl rat versus BTZ-treated rat colocalization degree: ^§^ *P* = 0.090; ^§§^ *P* = 0.072; *P ≤ 0.05; **P = 0.0016.
