# Supplementary material for: Bortezomib Treatment Produces Nocifensive Behavior and Changes in the Expression of TRPV1, CGRP, and Substance P in the Rat DRG, Spinal Cord, and Sciatic Nerve
Source: Biomed Res Int. 2014 Apr 27;2014:180428. doi: 10.1155/2014/180428 (PMC4022313; doi:10.1155/2014/180428)
Supplement: Supplementary file 1 — Supplementary Figures 1 and 2, respectively, show TRPV1-, CGRP-, and SP-LI DRG neurons in control and acutely BTZ-treated rats and their relevant size frequency histograms. Supplementary Figure 3: shows double staining immunofluorescence for TRPV1 and either CGRP or SP in the DRG of control and acutely BTZ-treated rats. Supplementary Figure 4: shows immunoreactivity to TRPV1, CGRP, SP and relevant densitometry of immunostained sections in the dorsal horn of control and acutely treated rats. Supplementary Figure 5: shows the outcome of immunostaining for TRPV1, CGRP, and SP in sciatic nerve sections of control and acutely treated rats. [file 180428.f1.pdf]

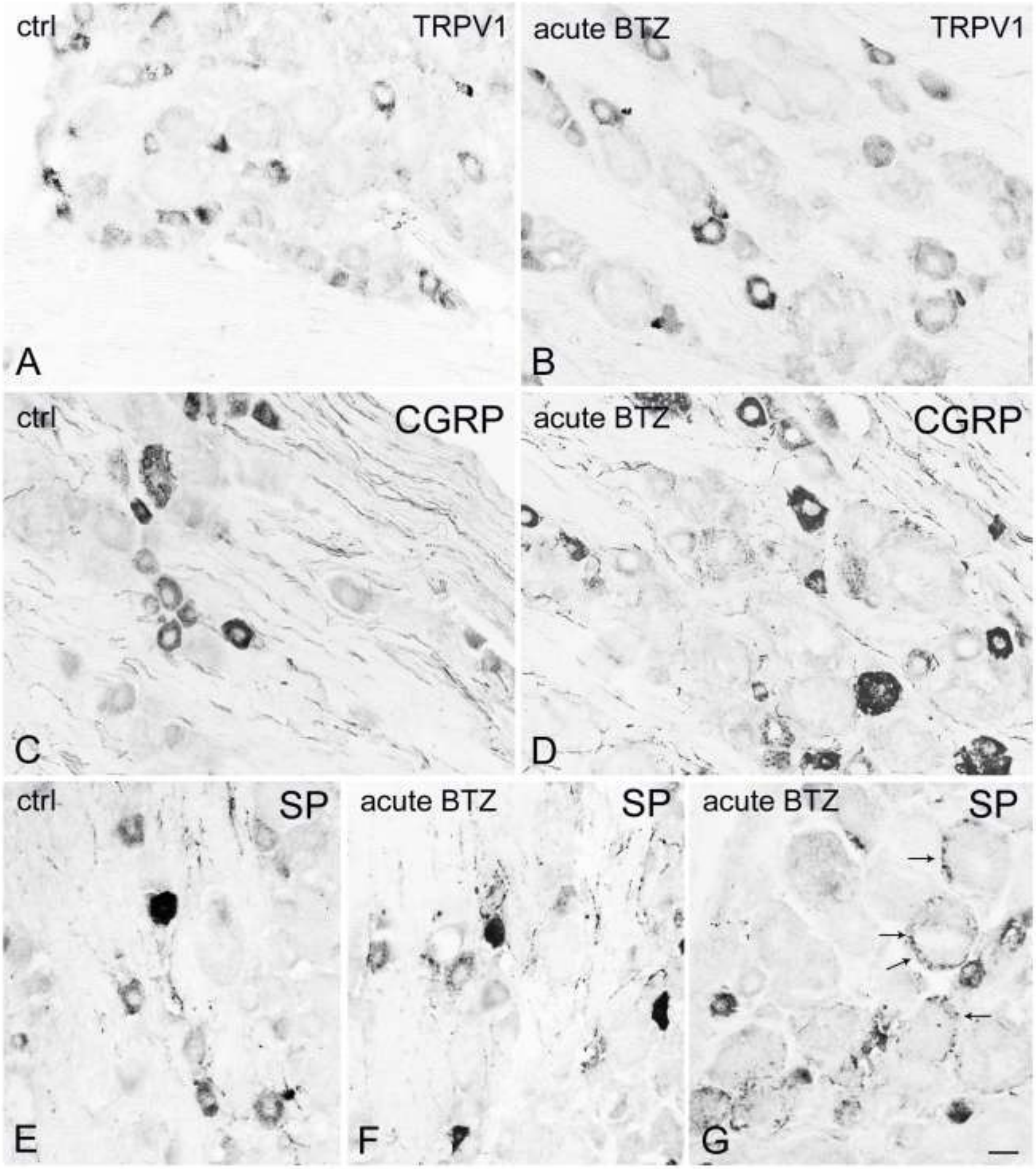

Supplemental Figure 1

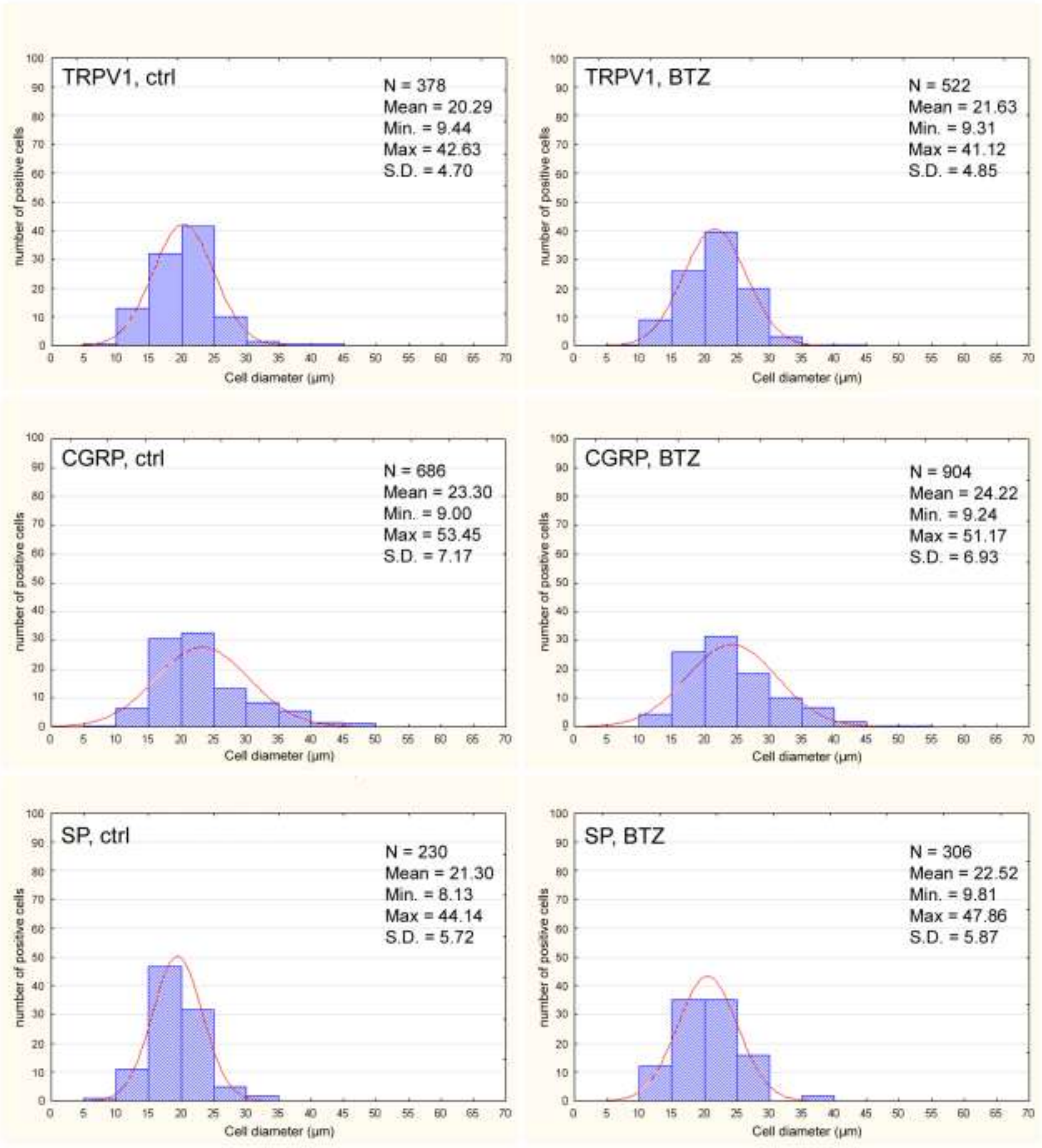

Supplemental Figure 2

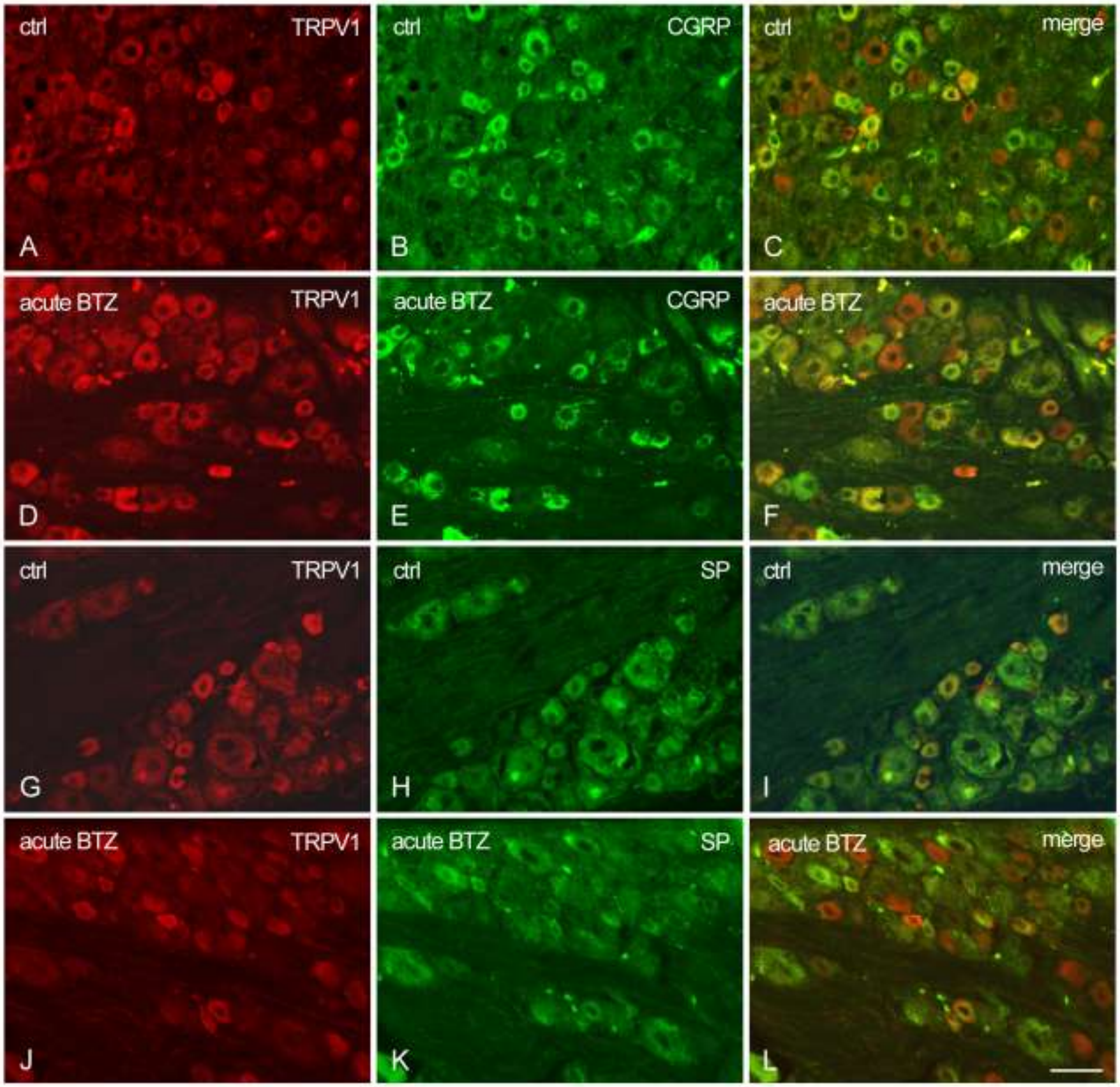

Supplemental Figure 3

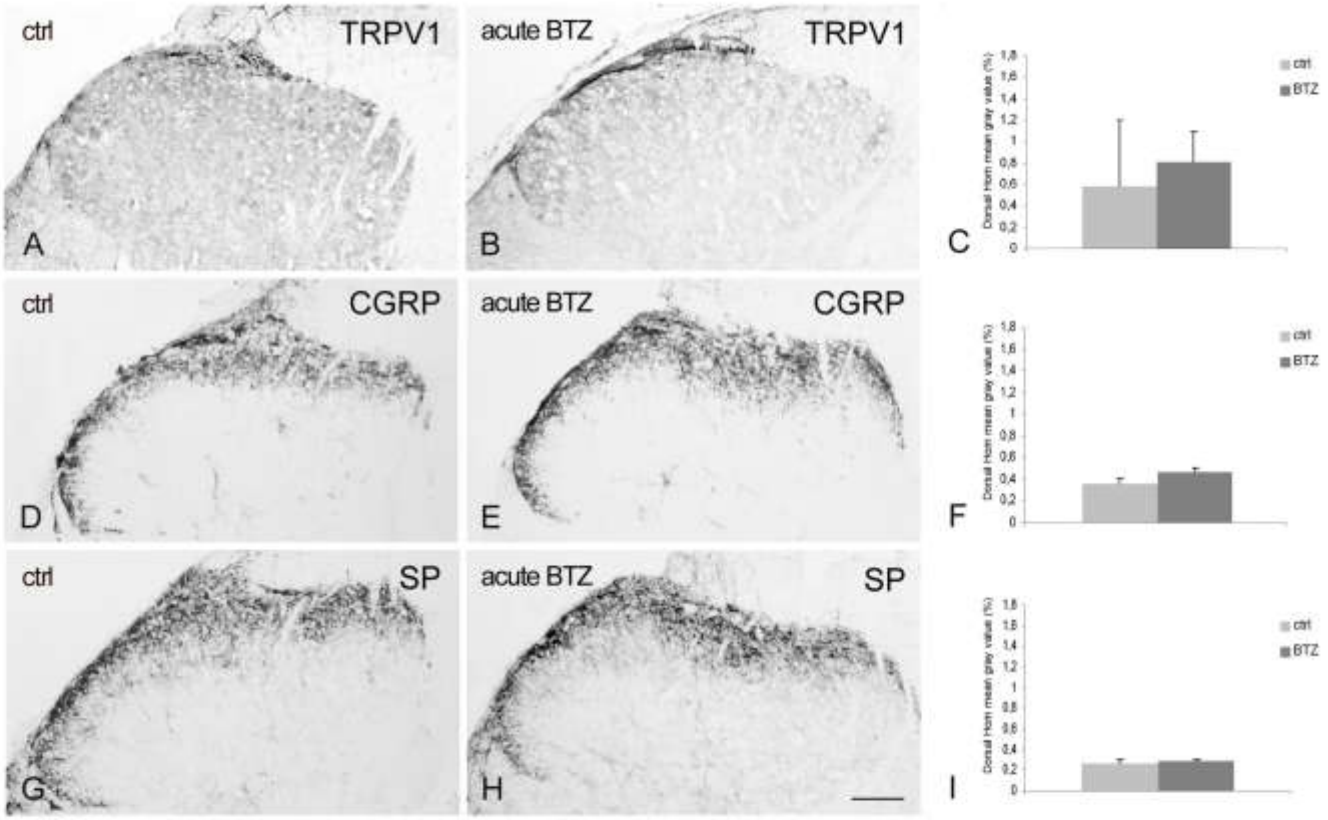

Supplemental Figure 4

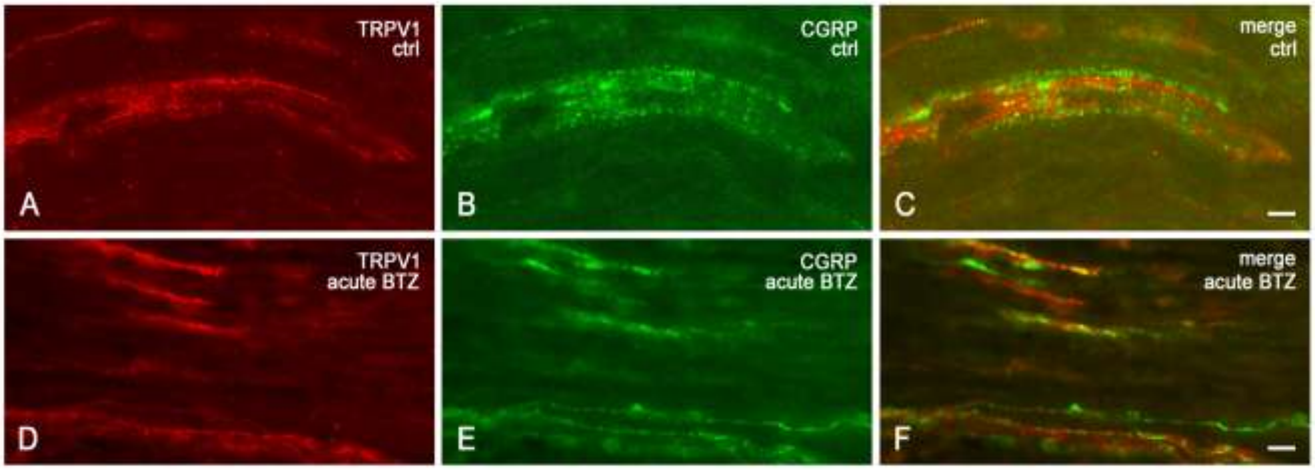

Supplemental Figure 5

Supplemental files related to this article

Supplemental Figure 1

Immunoreactivity to TRPV1 (A, B), CGRP (C, D) and SP (E-G) in representative sections of lumbar DRG from control (ctrl) (A, C, E) and acutely BTZ-treated rats (B, D, F, G). Arrows in G point to SP-positive satellite cells. Scale bar = 25 µm.

Supplemental Figure 2

Size frequency histogram of TRPV1-, CGRP-, and SP-LI DRG neurons from control (ctrl) and acutely BTZ-treated rats**.** Cells present in at least 6 sections were measured. x-axis values represent the mean cell diameters expressed in µm; y-axis reports values of relative percent frequency. Curve superimposed on the histogram represents the theoretical normal distribution. N = total number of sized positive neurons; S.D. = standard deviation.

Supplemental Figure 3

Double labeling immunofluorescence for TRPV1/CGRP (A-C, D-F) and TRPV1/SP (G-I, J-L) in DRG neurons from control (ctrl) (A-C, G-I) and acutely BTZ-treated rats (D-F, J-L).. C, F, I, L represent the composite images obtained by overlay of A-B, D-F, G-I and J-L, respectively. Scale bar = 25 µm.

Supplemental Figure 4

Immunoreactivity to TRPV1 (A, B, C), CGRP (D, E, F) and SP (G, H, I) in representative sections of lumbar spinal cord dorsal horn from control (ctrl) (A, D, G) and acutely BTZ-treated rats (B, E, H). Scale bar = 100 µm.

Supplemental Figure 5

Double labeling immunofluorescence for TRPV1/CGRP in sciatic nerve from control (ctrl) (A-C) and acutely BTZ-treated rats (D-F). C, F represent the composite images obtained by overlay of A-B and D-E, respectively. Scale bar = 10 µm.
